# Nanobiotics against antimicrobial resistance: harnessing the power of nanoscale materials and technologies

**DOI:** 10.1186/s12951-022-01573-9

**Published:** 2022-08-12

**Authors:** Nayanika Chakraborty, Diksha Jha, Indrajit Roy, Pradeep Kumar, Shailendra Singh Gaurav, Kalisvar Marimuthu, Oon-Tek Ng, Rajamani Lakshminarayanan, Navin Kumar Verma, Hemant K. Gautam

**Affiliations:** 1 Department of Chemistry, University of Delhi, New Delhi, 110007 India; 2 Department of Immunology and Infectious Disease Biology, CSIR-Institute of Genomics and Integrative Biology, Sukhdev Vihar, New Delhi, 110025 India; 3 Academy of Scientific and Innovative Research (AcSIR), Ghaziabad, 201002 India; 4 Department of Genetics and Plant Breeding, Faculty of Agriculture, Chaudhary Charan Singh University, Meerut, 250004 India; 5 National Centre for Infectious Diseases (NCID), Singapore, 308442 Singapore; 6 Ocular Infections and Anti-Microbials Research Group, Singapore Eye Research Institute, The Academia, 20 College Road, Singapore, 169856 Singapore; 7 Department of Pharmacy, National University of Singapore, Singapore, 117543 Singapore; 8 Academic Clinical Program in Ophthalmology and Visual Sciences Academic Clinical Program, Duke-NUS Medical School, Singapore, 169857 Singapore; 9 Lee Kong Chian School of Medicine, Nanyang Technological University Singapore, Clinical Sciences Building, 11 Mandalay Road, Singapore, 308232 Singapore; 10 National Skin Centre, Singapore, 308205 Singapore; 11 CSIR-Institute of Genomics and Integrative Biology, Mall Road, Delhi University Campus, 110007 New Delhi, India; 12 Tan Tock Seng Hospital (TTSH), 308433 Singapore, Singapore

**Keywords:** Antibiotics, Antibiotic resistance, Bacteria, Nanotechnology, Nanomaterials

## Abstract

Given the spasmodic increment in antimicrobial resistance (AMR), world is on the verge of “post-antibiotic era”. It is anticipated that current SARS-CoV2 pandemic would worsen the situation in future, mainly due to the lack of new/next generation of antimicrobials. In this context, nanoscale materials with antimicrobial potential have a great promise to treat deadly pathogens. These functional materials are uniquely positioned to effectively interfere with the bacterial systems and augment biofilm penetration. Most importantly, the core substance, surface chemistry, shape, and size of nanomaterials define their efficacy while avoiding the development of AMR. Here, we review the mechanisms of AMR and emerging applications of nanoscale functional materials as an excellent substitute for conventional antibiotics. We discuss the potential, promises, challenges and prospects of nanobiotics to combat AMR.

**Graphical Abstract:**

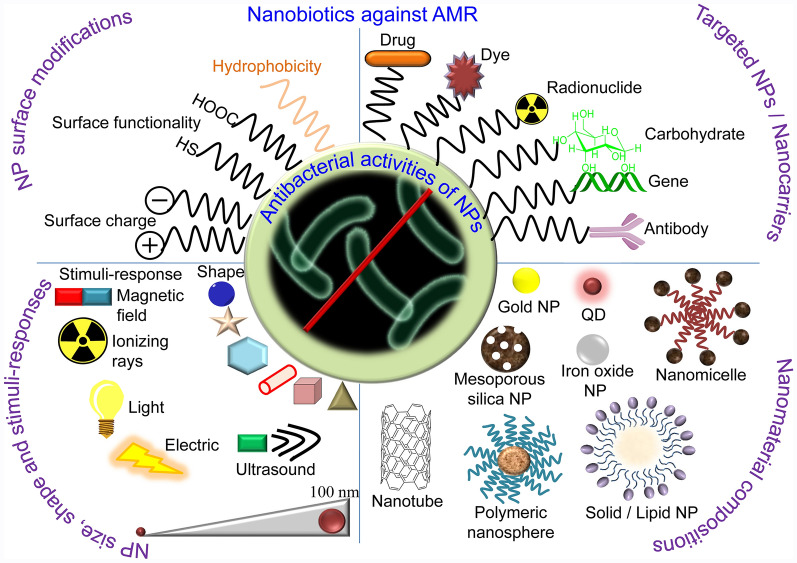

## Introduction

**Fig. 1:**
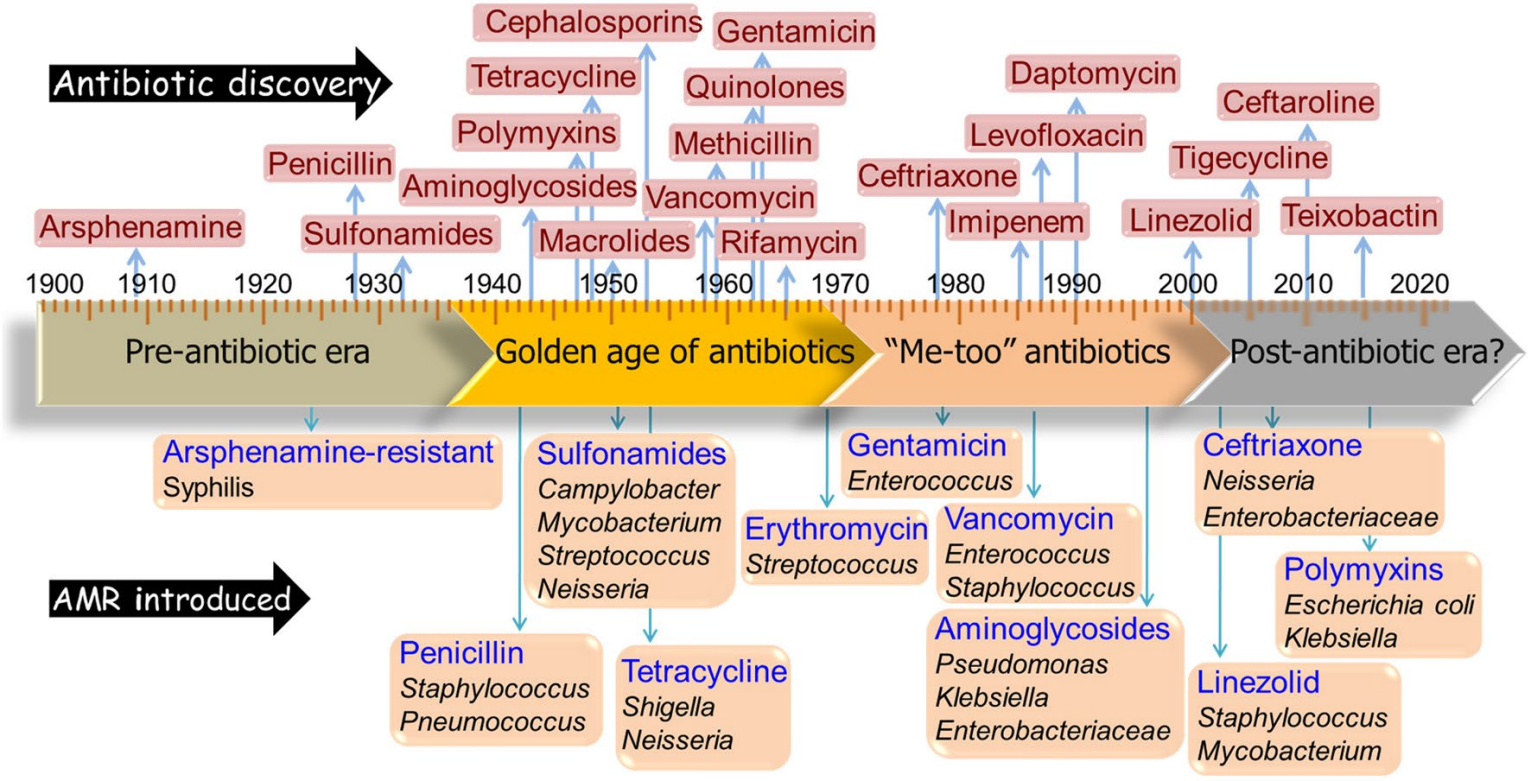
Timeline depicting the discovery of major antibiotics and subsequent emergence of resistance against them in various bacterial strains. One of the most dramatic events in the field of microbiology was the commercialization of penicillin in mid-1940s during the beginning of the industrial antibiotic era. While millions of human lives have been saved since then, the number of AMR cases continues to increase. AMR bacteria and their genes now circulate among humans, livestock, wildlife, environment, wastewater, and soil

Antibiotics have revolutionized modern medicine for treating bacterial infections. However, indiscriminate use, misuse and often abuse of antibiotics over time have led to rapid emergence of pathogenic strains with antimicrobial resistance (AMR). These bacterial strains can escape conventional treatment modalities. It is disappointing to realize that the development of AMR has outpaced the discovery of new antibiotics (Fig. 1).

Data from 204 countries and territories revealed that drug-resistant bacterial infections claimed 4·95 million people in 2019 and 1·27 million deaths were directly caused by AMR [1]. With current COVID-19 outbreak, this number is estimated to escalate up as more patients with viral infection are administered with antibiotics to treat secondary infections [2]. Thus, there is an urgent need to tackle AMR with new and innovative approaches. In this context, nanotechnology has opened-up avenues to deal with AMR.

Nanomaterials offer opportunities to access antibacterial modalities novel to bacteria that do not fall in their natural defence arsenal. The therapeutic effect of nanomaterials is largely derived from nanoscale confinement of materials compounded with multivalent interactions and high surface-to-volume ratio. Nano-size metals, metal oxides, organic nanoparticles (NPs), and nanocomposites with potent antibacterial activities are strategically advantageous to safely control superficial infections and infectious diseases. Diverse chemical compositions and intrinsic properties of these antibacterial nanomaterials (or nanobiotics) render multifaceted modes of action against the target bacteria. Notably, physiological states of the bacteria, i.e., planktonic, biofilm, stationary, starved, and logarithmic growth phase, impact their sensitivity to specific nanomaterials. Factors such as aeration, pH, temperature, and many other environmental features greatly influence antimicrobial activities of nanomaterials. These properties of nanobiotics offer opportunities to access unique mechanism of action that selectively and effectively target the bacterial systems.

**Fig. 2:**
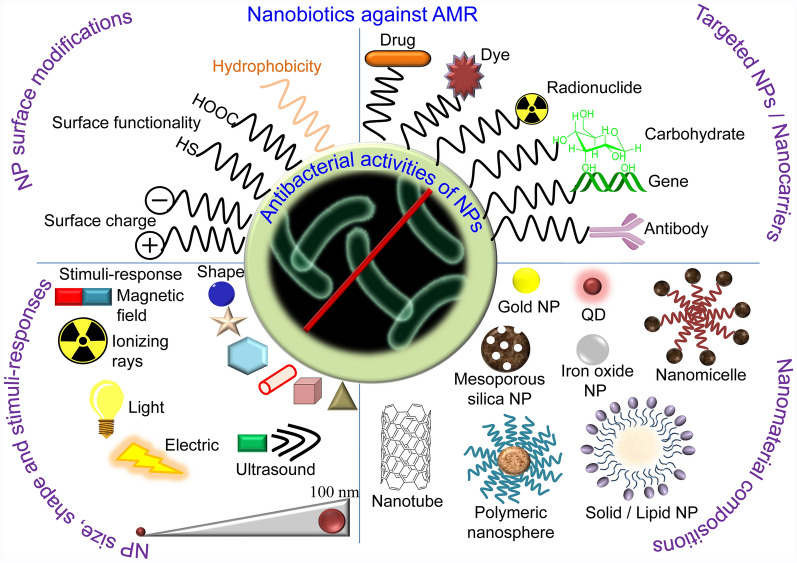
Antibacterial nanostrategies. NPs can complement and back antibiotics as a good carrier. The unique small size of nanomaterials grades in novel properties, such as increased interaction with bacteria due to larger surface area-to-mass ratio, and versatile plus controllable applications. Efficacy of the NPs can be increased by tuning sizes, shapes, and chemical compositions of the NPs. Metallic, organic, biomolecular, radio- and antibody modified NPs can effectively distroy bacteria with multiple mechanisms and their potency can be enhanced with addition of ultrasound, magnetic field, light, and ionizing radiation properties

Here, we review the AMR and provide a comprehensive evaluation of the state-of-art nanotechnology to address the problem of AMR, emphasizing the mechanism of actions of nanobiotics (Fig. 2). We discuss associated challenges of antibiotic discovery as well as prospects of functional nanomaterials as vaccines.

## Epidemiology and etiology of AMR

In 2010, BRICS countries (Brazil, Russia, India, China, and South Africa) contributed to 76% antibiotic consumption, with 12.9 billion units consumed by India, and 10 billion units by China [3]. As of 2017, carbapenem-resistant *Acetobacter baumannii* and *Enterobacteriaceae* burdened about US$281 million to the US healthcare costs [4]. In general, hospital costs increase significantly for treating patients with AMR infections due to the requirements of higher doses of drugs and longer hospital stays. Moreover, hospital-acquired infections with ESKAPE pathogens (*Enterococcus faecium*, *Staphylococcus aureus*, *Klebsiella pneumonia*, *A. baumannii*, *Pseudomonas aeruginosa*, and *Enterobacter* species) further complicate the treatment. It is anticipated that the global cost for treating AMR can reach up to US$100 trillion by 2050 [5]. The Centers for Disease Control and Prevention (CDC), USA, list of emerging AMR threats on the basis of level of urgency is summarized in Table 1.

**Table 1:** Antimicrobial resistance threat data as reported by CDC in 2019

Level of concern to human health	Bacteria	Approximate number of deaths/year (year)
Urgent threat	Carbapenem-resistant *Acinetobacter baumannii*	700 (2017)
	*Clostridioides difficile*	12,800 (2017)
	Carbapenem-resistant *Enterobacterales*	1100 (2017)
	Drug-resistant *Neisseria gonorrhoeae*	550,000 infections/year (2017)
Serious threat	Drug-resistant *Campylobacter*	70 (2017)
	Extended spectrum β-lactamase (ESBL)-producing *Enterobacterales*	9100 (2017)
	Vancomycin-resistant *Enterococcus*	5400 (2017)
	MDR *P. aeruginosa*	2700 (2017)
	Drug-resistant nontyphoidal *Salmonella*	70 (2017)
	Drug-resistant *Salmonella serotype Typhi*	< 5 (2017)
	Drug-resistant *Shigella*	< 5 (2017)
	Methicillin-resistant *Staphylococcus aureus (MRSA)*	10,600 (2017)
	Drug-resistant *Streptococcus pneumonia*	3600 (2014)
	Drug-resistant *Tuberculosis*	62 (2017)
Concerning threats	Erythromycin-resistant Group A *Streptococcus (GAS)*	450 (2017)
	Clindamycin-resistant group B *Streptococcus (GBS)*	720 (2016)
Watch list	Drug-resistant *Mycoplasma genitalium*	–
	Drug-resistant *Bordetella pertussis*	–

## Genesis of AMR

Extensive research in the recent past has discovered several illuminating mechanisms of AMR. These include crucial roles of gene mutations, genetic linkage, biochemical compositions in the bacteria, and efflux pumps as described below.

### Genetic mechanism of AMR

The prolonged non-judicial usage of antibiotics in clinics and non-clinical use (e.g., in farm animals, aquaculture, poultry, meat, and plants) have eroded their therapeutic efficacy and resulted in increased number of AMR bacterial strains [6]. The selection pressure causes de novo evolution of resistant strains through a stochastic process. A sensitive cell undergoes genomic mutations or acquires gene(s) for resistance through horizontal gene transfer (HGT) using plasmids or phages. The newly acquired resistant cell survives, divides into large number of resistant daughter cells, and establishes as an AMR strain [7]. For example, introduced as the most potent antibiotic, penicillin was widely used for treating patients with Staphylococcal infections. However, soon after its inception, these bacteria developed β-lactamase, the penicillin deactivating enzyme [6]. *Streptomyces* and *Micromonospora* acquired resistant genes that produce their own antimicrobial product (aminoglycosides) [6]. Many *Enterococci* and *Mycoplasma* species contain the plasmid encoding the gene *tetM*, responsible for resistance to tetracycline. OtrB and OtrC genes in *Streptomyces rimosus* express efflux proteins that provide self-resistance to these bacteria [6]. Multi-drug resistant (MDR) phenotypes of *Shigella* have been transferred to *Escherichia coli* showing rapid spread of resistance genes in a pool of bacteria [8, 9], further confirms AMR genomic drivers and HGT.

### Membrane remodelling mechanism of AMR

**Fig. 3:**
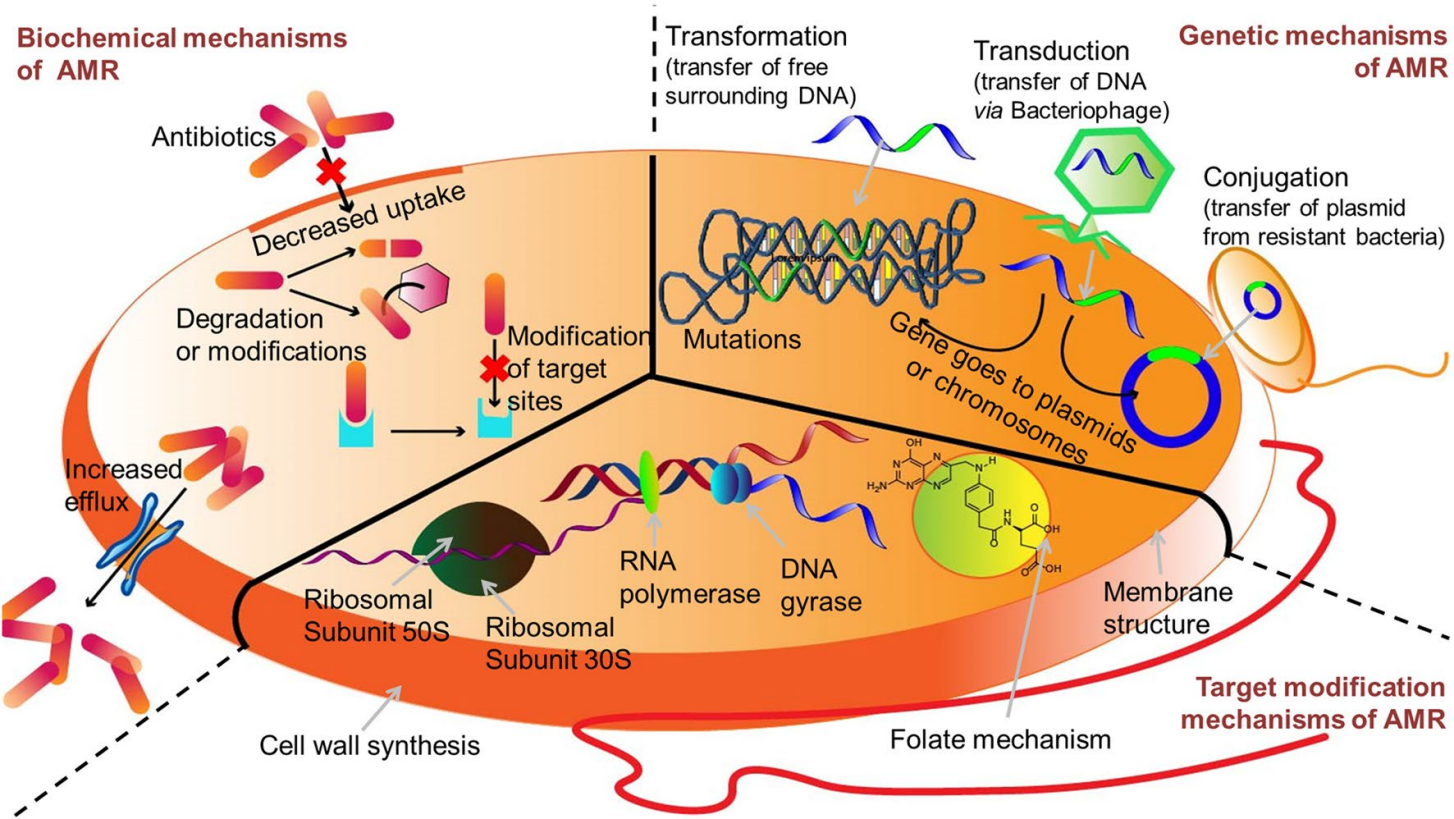
The depiction of defense arsenal mechanisms of resistance in bacteria against conventionally used antibiotics. The presence of antibiotic resistance elements in pathogens has made AMR more challenging because of prevalence of HGT. Certain bacterial species are inherently resistant to antibiotics because of an impermeable membrane or lack of the antibiotic targets. Few have MDR efflux pumps that remove antibiotics from the bacteria. Some microbes possess altered genes, target protein, disabling the antibiotic-binding site(s). Inactivation of antibiotic can occur by covalent modification of antibiotics. The AMR mechanism in Gram-positive and Gram-negative bacteria can be different because of morphological and structural differences

By virtue of complexity and diversity of cell surface layers, bacteria can limit the access of antimicrobials at the site of action by multiple mechanisms (Fig. 3). For example, they utilize β-lactams, aminoglycosides, and energy dependent efflux to prevent the accumulation of tetracycline [6]. Outer membrane (OM) remodelling with synchronization of reduced drug uptake and increased active drug efflux facilitate the multiplicative actions of OM in developing MDR [10].

The cell envelop architecture of the Gram-positive bacteria is simpler than that of Gram-negative bacteria comprising of a cell membrane surrounded by a thick peptidoglycan cell wall. Despite the absence of OM, modification of peptidoglycan or lysinylation of phosphatidylglycerol in *S. aureus* decreases the cellular entry of antibiotics with intracellular targets, such as hydrophilic fluoroquinolones. The decreased drug intake can directly contribute to resistance or/and in conjunction with the overexpression of efflux pumps that has been observed in the development of *P. aeruginosa* imipenem resistance in patients with bloodstream infections in Taiwan [11]. The expulsion of drugs is facilitated by a series of conformational changes in the periplasmic accessory protein AcrA that brings AcrB and To1C in close proximity. This causes the ejection of substrate by To1C from the cytoplasm to exterior of the cell [12]. For example, fluoroquinolone-resistant *E. coli* overexpresses AcrAB proteins that confer them with high level of resistance against fluoroquinolones [13]. Increased efflux of chlorhexidine and other biocides through the efflux pump AcrAB-TolC has been identified as an important mechanism of AMR in *Klebsiella* spp. [14]. Tetracycline efflux pumps encoded by *tetA*, *tetB,* and *tetK* genes acquired through HGT provide resistance to tetracycline in both Gram-negative and Gram-positive bacteria [15].

### Target modification mechanism of AMR

Antibiotic-target interaction is very specific, and any alteration of the target can reduce the antimicrobial activity. Mutations can alter clinically important targets by multiple mechanisms. These include (i) modification or aberrant enzyme production for folate pathway, transcription, and DNA replication, (ii) reduction in binding affinity of essential cell wall synthesis enzymes, in case of β-lactams or cell wall precursor modification, (iii) changes in ribosomal proteins 16S RNA, and (iv) dimethylation of 23S ribosomal RNA. Modifications of enzymes involved in folate pathway confer “bypass” resistance to sulphonamide and trimethoprim [16].

Mutation in the *dhfr* gene causing single amino acid substitution in the target enzyme, dihydrofolate reductase, leads to trimethoprim resistance in *S. aureus* and *S. pneumoniae* [17, 18]. Alterations in DNA gyrase (*gyrA* and *gyrB* genes) and topoisomerases (*parC* and *parE* genes) bacterial sensitivity to fluoroquinolones (ciprofloxacin, norfloxacin) [19, 20]. Mutations, insertions, or deletions of amino acids residues in a small fragment of the *rpoB* gene result in rifampicin-resistance in *M. tuberculosis* and *E. coli* [21]*.* Resistance to methicillin, oxacillin and cephalosporins in *S. aureus* is credited to the expression and acquisition of the *mecA* encoding PBP2a, which has reduced affinity for β-lactam antibiotics [22]. Sporadic increase in resistance against glycopeptides in *E. faecium* and *E. faecalis* isolates is attributable to the acquisition of *van* gene clusters, which encode for enzymes ligase, dihydrogenase, and serine racemase [23]. The *vanA* gene clusters also confer high-level resistance to vancomycin and teicoplanin [24].

Target site for the binding of macrolide, ketolids, lincosamide, streptogramin B (MKLS_B_) is the large 50S subunit of ribosome consisting of 5S, 23SrRNA and 33 proteins. The MKLS_B_ type resistance is mediated by *erm*-encoded rRNA methyltransferases, catalysing a post-transcriptional modification of 23S rRNA. Methylation or dimethylation of key adenine bases (e.g., A2058 in *E. coli*) of domain V within 23S rRNA alters the site of antibiotic binding to ribosomes [25]. High-level linezolid resistance in *Clostridium* spp. is attributed to *Cfr*-mediated methylation of 23S rRNA [26].

### Drug inactivation mechanism of AMR

The problem of AMR is exacerbated by enzymes that can irreversibly destroy antibiotics. For example, β-lactamase mediated resistance involves the kinetic interaction and subsequent hydrolysis of ester or amide bond of β-lactam substrate, before reaching the site of action. Aminoglycoside-modifying enzymes, such as aminoglycoside phosphotransferases, aminoglycoside acetyltransferases, and aminoglycoside nucleotidyltransferases, catalyse the modifications of amino or hydroxyl group of aminoglycosides (e.g., tobramycin, amikacin, isepamicin, and sisomicin), interfering their antibacterial potential by impeding its binding to the 30S ribosomal subunit [27].

Chloramphenicol resistance is attributed to chemical modification by chloramphenicol acetyl transferases, which acetylate chloramphenicol rendering it incapable of inhibiting bacterial protein synthesis [28]. Flavin-dependent monooxygenase TetX inactivates tetracyclines, which is attributed to mono-hydroxylation of tetracycline resulting in intramolecular cyclization and degradation of the antibiotic. Streptogramin acetyltransferase present in *Staphylococci* and *Enterococci* species acetylates the unbound hydroxyl group of streptogramin A and thus inactivates it [29].

### Metabolic alteration mechanism of AMR

Antibiotics affect bacterial metabolism in a complex way. Mutations in the 2-oxoglutarate dehydrogenase (*sucA*) were found in genomes of > 3500 clinical *E. coli*, implying their role in AMR. Undeniably, metabolic adaptation might embody a discrete class of resistance mechanism beyond conferring tolerance, when cells also amend their metabolic response to palliate downstream toxicity caused by antibiotics [30].

### Plasmid-host co-evolution mechanism of AMR

Plasmid-host co-evolution occurs through (i) mutations linked to plasmid DNA replication and host which compensate the interference cost of plasmid, (ii) gaining of a native transposon which carries the toxin–antitoxin system, and (iii) compensatory mutations in the global regulatory pathway of the host. Parasitic plasmid-host association when antibiotics are absent in the environment evolves into mutualism when both bacteria and humans require the presence of antibiotics to survive. This accentuates that plasmid-bacteria pairs are evolving on a rocky adaptive landscape with various evolutionary trajectories, and clonal interference plays a vital role in plasmid-host co-evolutionary dynamics [31].

### Antibiotic tolerance mechanism of AMR

There has been increasing incidence where bacterial populations adapted and eventually became tolerant and resistant to the antibiotic [32]. Antibiotic tolerance can be a result of genetic mutation but also arises due to phenotypic factors associated with traumatic environment, such as low temperature, serum levels, pH, host factors, ionicity, size and/or growth phase of inoculum and antibiotic exposure [33, 34]. Antibiotic persistence is observed in a small subpopulation of bacteria (< 1%) that can survive lethal concentrations of the antibiotics [35]. Moreover, current diagnostic systems fail to rapidly detect specific pathogens and delay in diagnosis can lead to AMR development in pathogens. *M. tuberculosis* is the most antibiotic tolerant bacteria of clinical relevance due to its stochastic and excessively slow growth in the lung. Transition of *E. coli* between walled (susceptible) and cell wall deficient (tolerant) forms to survive cell wall inhibiting antibiotic has been encountered in patients with high level of recurrence in urinary tract infections (UTIs) [35, 36].

### Biofilm formation mechanism of AMR

Biofilms provide considerable survival advantage to pathogenic bacteria and is responsible for recalcitrant infections especially associated with medical devices (catheters, sutures, prosthetic devices, implants, cardiac valves, intrauterine contraceptive devices etc*.*), skin and soft tissues, endocarditis UTIs and otitis media. Biofilms account for 65–80% of bacterial infections [37]. A biofilm can be described as sessile microbial community composed of one or more species that colonize and irreversibly attach on a surface or to each other encased in an extracellular matrix This extracellular matrix is composed of extracellular polymeric substances (EPS) that include proteins, carbohydrates, glycoproteins, glycolipids, and nucleic acids from biofilm forming bacteria as well as the host [38]. EPS matrix of biofilms provides a physical barrier that limits antibiotic penetration and imparts AMR development by multiple mechanisms. For example, sub-inhibitory antibiotic dosing reaching the colonized bacterial population of biofilm aids in antibiotic tolerance. The glycocalyx layer of EPS serves as an adsorbent for exoenzymes that inhibit the transportation of antibiotics. Biofilms add in the accumulation of enzymes that covalently modify antibiotics, deactivating and impeding their antibacterial potency [39]. In addition, anionic components and extracellular DNA present in the EPS matrix serve as cation chelators, generating a cation-deficient environment and thus promoting AMR [40].

The biofilm formation promotes intra-species associations leading to heterogeneity and subsequently, antibiotic tolerance in polymicrobial biofilms. The nutrient and oxygen concentration gradient across EPS matrix causes bacterial population with varying growth rates. Superficially located bacteria consume surface obtainable oxygen and nutrients before it disperses into the depths of biofilm. Since, antibiotics are more lethal towards metabolically active cells, the deeply located tolerant cells escape the insult.

### Swarming motility contributes to AMR

Swarming motility is a form of social behaviour of bacteria that aids to the rapid coordinated movement of bacterial cells on a moist surface. Many bacterial species demonstrate non-genetic adaptive resistance to a broad range of antibiotics during swarming. For example, *P. aeruginosa* PA14 is more resistant to aminoglycosides, chloramphenicol, ciprofloxacin, β-lactams, macrolides, tetracycline, and trimethoprim under swarming conditions [41]. In contrast to slow metabolic state of AMR species, bacterial swarms are metabolically active and grow vigorously so a new terminology STRIVE (swarming with temporary resistance in various environment), draws a difference between swarming specific adaptive resistance from growth-related resistance. A plausible mechanism involves the release of a necro signal from dead cells that activates the antibiotic survival for the swarmer cells [42].

### Host intracellular environment in AMR

Antibiotic tolerance is common in various obligate and facultative pathogens, such as *Mycobacterium* species, *K. pneumonia*, *Neisseria*, *Legionella*, *Brucella*, *Francisella*, *Listeria*, *Salmonella*, *Rikettsia*, *Ehrlichia*, *Coxiella*, and *Chlamydia* [43, 44]. These pathogens survive by either membrane bound or cytosolic environment eventually escaping not only antibiotic exposure but also the host immune defences. Thus, infections caused by intracellular pathogens are difficult to treat since conventional antimicrobials are unable to infiltrate. For example, *K. pneumoniae* and *E. faecalis *are able to survive and thrive within intracellular vacuolar compartments upon engulfment by alveolar macrophages [45, 46].

## AMR and challenges in traditional antibiotic discovery

Earlier research during 1990s gave birth to bacterial genomics that bared a horde of antibacterial targets, revealing over hundred novel antimicrobial mechanisms. However, companies like Pfizer, AstraZeneca, and GlaxoSmithKline are yet to come up with a novel candidate. Challenges are evident as no Gram-negative bacterial antibiotic with an entirely novel mechanism has been approved in the past 40 years [47]. Most leads could not be conceived into a hit having balance of desired antibacterial, pharmacokinetic, and safety profiles. A large fraction (> 90%) of the identified hit molecules were not suitable for treating AMR infections, whereas the remaining molecules had either low potency against MDR bacteria or were toxic for clinical use [48]. Reasons for the poor success rate in antibiotic development include (i) scientific challenges to discovering new classes of antimicrobial agents, mainly due to insufficient knowledge about AMR origin, diversity and mechanisms, limited understanding of novel targets, technical difficulties in hit-to lead selection, and undesirable pharmacological and safety profiles, (ii) obstacles in conducting clinical trials, such as identifying and recruiting patients, (iii) high competition from approved and currently used antibiotics, (iv) anticipated risk that new antibiotic may become ineffective within a couple of years due to possible resistance development, and (v) requirement for substantial investment during the preclinical research and clinical trials coupled with low approval ratings, numerous challenges on market entry, and much lower profitability than non-antibiotic drugs [48–50]. As of 2017, the average cost of developing an antibiotic was estimated to be about US$1.5 billion [51].

Another major challenge for antibiotic discovery is the ubiquitous presence of AMR genes in the ecosystems. The human intestinal microbiota is a reservoir of MDR genes [52]. AMR genes exist in wastewater that supports the selection of MDR bacteria [53]. These genes have been acquired by many bacterial species that have empowered them to tolerate traditional antibiotics from insects, fungi, plant products, petroleum chemicals, agricultural and industrial wastes [54]. In this context, several questions remain a mystery—are these AMR genes there for discrete genetic/biochemical demands? Do bacteria show resistance because they are exposed to numerous toxins in the environment? How do natural products affect the ecology? Does presence of selective ecosystem pressure lead to resistance in strains? Hence, to stop the propagation of AMR in the environment, scientists must come up with innovative/designer antibiotics that tackle new and increasing AMR, generate less resistance, reduce the risk for cross-resistance, and are self-destroyed post treatment.

Most traditional antibiotics, which are natural or semisynthetic products, are plagued by significant shortfalls, lack of desirable antibacterial efficacy, development of AMR, and challenges associated with monitoring/evaluating antimicrobial activities in a dynamic milieu. They have failed to deliver activity against the most urgent threats from MDR Gram-negative bacteria. Research and development processes for discovering and developing a traditional antibiotic are time-consuming (about 10–12 years), expensive, and fraught with a multitude of barriers [49]. These antibiotics typically target essential processes of survival and/or growth of bacteria, synthesis and maintenance of cell wall/membrane, or DNA replication, transcription and/or translation. Unfortunately, bacteria can acquire the ability to escape the effects of these drugs, irrespective of target functionalities. Another challenge for traditional antibiotic discovery is lack of effective techniques for isolating and purifying naturally occurring antimicrobials against MDR bacteria. Treatment for MDR bacterial infection requires high dosages of traditional antibiotics, often cocktail of multiple drugs or the ‘last resort’ antibiotics. Adding to the therapeutic burden is biofilm-associated resistance, which requires exceptionally high dosages of antibiotics due to difficulty in penetrating the extracellular polysaccharide sheath covering the biofilm and the presence of complex microflora. High dosages of traditionally used antibiotics often result in long and expensive treatment with serious side effects and uncertain outcomes. Furthermore, the utilization of conventional antibiotics carries a high-risk alert for AMR development.

A critical analysis of traditional antibiotics that were approved during 1999–2014 showed lack of novelty and diversity for target bacteria. These antibiotics failed to address AMR issues. It was suggested to apply therapeutic alternatives to antibiotics that would decrease our dependence on traditional antibiotics. The fact that antibiotic effectiveness is reduced over time due the potential development of resistance, new antibiotics will always be needed [55].

## Nanotechnology to rescue AMR issues

Emerging nanoscale materials and technologies provide long-lasting answers to the above issues due to the unique mechanism of action of nanomaterials on pathogenic bacteria. The “antibiotic nanocarriers” based on liposomal, solid/lipid, terpenoid, polymeric, dendrimeric, and inorganic materials have shown encouraging results in enhancing the overall antibiotic performance compared to bulk use of antibiotics [56]. This has been achieved by improving the pharmacokinetics of the drug—extending antibiotic half-life in the serum and decreasing the apparent volume distribution attributed to target the site of infection and causing elimination of bacteria even at lower drug dosage. The “combinatorial” or synergistic effect of either multiple drug entrapment in a single nano-construct or two or more NPs constructing hybrid NPs reinforces the pharmacological effects and improves the antibacterial potency while limiting the development of resistance. The modes of actions of nanomaterials to fight AMR strains are novel and unique that are not present in bacterial arsenal of natural defence (Box 1). In the following section, we discuss various types of nanomaterials and nanotechnologies that are being employed or currently investigated to tackle AMR.

Box 1: Essential parameters utilized by NPs to mount antibacterial actions

**Table:** 

Physicochemical properties of NPs including surface morphology, size, crystal structure, charge and zeta potential regulate their antibacterial actions. Bacterial strains, exposure time and environmental conditions also impact the potency of antimicrobial drugs. Some of the crucial factors that govern the antibacterial mechanism of NPs are summarized below ***Size* and *surface charge*** of NPs affect the antimicrobial potential. Length and breadth of nanotubes can extend the release of given antimicrobial. Large specific surface area of NPs increases the prospect of closeness, contact, and interaction with microbial membrane [56, 57] ***Shapes*** of nanomaterials are the basis of wavering degrees of damage to pathogens through periplasmic enzymes. For example, ZnONPs of various shapes (sphere, pyramid, and plate) exhibit varied photocatalytic activities with β-galactosidase leading to functional and conformational change in the enzyme [58]. Prismatic-shaped Y_2_O_3_ NPs show better activity against *P. desmolyticum* and *S. aureus*, which may be due to direct interaction of Y_2_O_3_ NPs and bacterial cell membrane surfaces, causing leakage of the cellular components [59]	***Roughness*** of NPs can decrease the adhesion of microbes, as the size and surface area-to-mass ratio stimulate the adsorption of bacterial proteins [60] ***Zeta potential*** of NPs resiliently impacts microbial adhesion because of electrostatic attraction generation due to negative charge on bacterial membrane and positively charged NPs [61]. For example, Mg(OH)_2__NPs, being positively charged are adsorbed on microbial surfaces and this accumulation at the site of infection ascends the permeability [62] ***Doping modifications*** allow proper dispersal of NPs in hydrophilic or aqueous environments. For example, nanocomposites framed with a combination of Au and ZnO show enhanced photocatalytic property and ROS formation as Au allows enriched light absorption (Surface Plasmon Resonance) and ZnO with transformed band gap width increases electron transport efficiency and charge carrier separation [63]. Cr doping on the ZnONPs significantly enhanced their antimicrobial activity against a wide range of pathogenic bacteria [64] ***Environmental conditions*** impact the antimicrobial potency of NPs. For example, most bacterial enzymes cannot function beyond optimum temperature and NPs targeting these enzymes would be ineffective. Reduced pH shows high dissolution of ZnONPs and adhesion of NPs on the bacterial membrane of MR*SA* and *E. coli* [65]

### Nanoscale antimicrobial substances

NPs can be classified into inorganic/organic, carbon-based, and hybrid structures. The inorganic group comprises of metal/metal oxide NPs and quantum dots (QDs). Organic nanomaterials are apt for drug delivery, antimicrobial use, bio-imaging, and tissue regeneration, as these consist of biocompatible organic components. This class of materials comprises of polymeric NPs, liposomes, and lipid-based NPs. Carbon-based nanomaterials embody carbon black, nanotubes, graphene, nanofibers, nanodots, fullerenes, nano-diamond, carbon onions, carbon rings, etc*.*

*Inorganic NPs* are made up of inorganic oxides of Si, Ag, Au, Zn, Mg, Mn, Cu, Se, Al, or Ti and hence they differ in shape, size, solubility and stability. Their characteristics are also defined by pH, temperature, reduction time, concentration of the reducing agent, and aggregation behaviour that impact their antimicrobial potency [66, 67]. For example, iron oxide NPs (FeONPs) are allied with DNA hybridization technique to heighten the capturing of 16S ribosomal RNA gene of bacteria [68]. Silver NPs (AgNPs) attach to cell membrane, interact with membrane proteins, increase membrane porosity, enter and enhance generation of reactive oxygen species (ROS) hampering respiration, including bacterial cell lysis, and evoking inflammatory reactions [69]. A recent study shows that the pH-triggered aggregation of AgNPs favors their penetration into bacterial biofilms [70]. The large aggregated AgNPs remain in the biofilms for a long time, disrupt bacterial biofilm formation, and exhibit better antibacterial activity than traditional AgNPs [70]. Gold NPs (AuNPs) exert bactericidal effects by accumulating on the cell surface credited to heavy electrostatic forces accompanied by cytoplasmic leakage and cell death [71]. Au nanocrystals display facet-dependent antibacterial activities—bacterial membrane damage, inhibition of cellular enzymatic activity, and energy metabolism [72]. QDs are metallic in nature but contain a core of semiconductor materials like Cd or Zn. The antimicrobial activity of QDs can be assigned to their ability to destroy bacterial cell walls or membranes, induce free radicals, bind with genetic material, and inhibit energy production [73]. Their sensitivity can further be increased by functionalization. For example, transferrin-modified silver QDs coupled with zinc and rifampicin exhibit much higher antibacterial activity than the zinc and rifampicin complexes [73]. QDs of custom sizes can be engineered to distinguish various mutants of same microbial species [74]. Photoexcited QDs have been found to inhibit growth of MDR clinical isolates (*S. typhimurium*, MRSA, *K. pneumonia*, and carbapenem resistant *E. coli*) due to redox potential of photogenerated charge carriers that interact with the bacterial environment [75]. Due to their high drug loading capacity and ability to cross barriers, these are employed for intracellular delivery of peptides, drugs and nucleic acids [76].

ZnO and CuO NPs exhibit admirable antibacterial properties but their accumulation has raised safety concerns in the host’s system. Human cells have Cu-transporters for regulation of Cu homeostasis, unlike Au and Ag for which such mechanism has not been reported yet [77]. CaF_2_ NPs have shown lethal effects against *S. mutans* due to their adherence on the tooth surfaces and persistent release of fluoride ions which stimulates re-mineralization and suppress virulent *S. mutans* [78]*.*

*Organic NPs* such as liposomes, polymeric micelles, polymeric and solid/lipid NPs constitute a class of organic NPs, which can carry both hydrophobic and hydrophilic antibacterial agents [79]. Most of these NPs are biocompatible and can be easily opsonized and degraded quickly due to their hydrophilic/hydrophobic individualities [79].

Liposomes and lipid NPs are spherical vesicles of phospholipid bilayers. They can fuse with microbial membrane and release the given drug into bacteria [80]. They have been developed into a drug delivery system for antimicrobial agents against biofilm-mediated infections. Their unique characteristics, including target-specificity, low toxicity, and ability to fuse biofilm matrix/cell membrane, enhance the efficacy of antibiotics by minimizing recurrent infections [80].

*Polymeric NPs*, such as nanospheres/nanocapsules, act as drug carriers. They are physically/chemically stable and enhance targeting efficacy. These NPs are prepared by polymerization of monomers (micro-/mini-emulsion) and from polymers via solvent evaporation, salting-out, and dialysis [81]. Lipid-based surface-functionalized PLGA is one such example of drug delivery system that protects antibiotics from degradation and has capability to bind to the components of biofilm [82]. Chitosan NPs can be loaded with antibiotics that exhibit super-antibacterial potential and are framed by ionic gelation of diverse concentrations [83]. Cellulose fibres reformed with AgNPs display sound antimicrobial defence against *E. coli* and *S. aureus* [84]*.* Graphene quantum dots (GQDs), AgNP and silica nano-fabrications enhance ROS production in light-activable GQDs, by conversion of light energy to hyperthermia, resulting in effective bacterial killing [85].

*Polymeric micelles* are colloidal structures made up of block copolymers. Polystyrene, poly(butyl methacrylate), polylactic acid (PLA), or poly(ethylene oxide), and poly(propylene oxide) have been used to form polymeric micelles [86]. The inner core of micellar structures is generally hydrophobic while outer core remains hydrophilic. These properties define drug release kinetics and charge determines the stability of micelles.

*Solid/lipid NPs* are lipid-based, solid, colloidal carriers like acetyl palmitate or salts of myristic acid. These are used for their nominal toxicity and stability. These particles show controlled release and high efficiency which differ by the nature of the lipids and milieu’s pH and temperature [87].

*Nanozymes* are nanomaterials with intrinsic enzyme-like properties [88]. FeO-based artificial peroxidase NPs have been used to treat drug-resistant *E. coli* and *S. aureus* [89]*.* Activity of nanozymes is superior because of the rough surfaces that allow bacterial adhesion, extremely irregular edges that act as active sites, ability to regulate ROS production and knack for photocatalytic activation [90]. In surface-bound state, nanozymes eliminate pathogens and delay the onset of resistance, while in the coated form, they can prevent biofilm formation [91].

*Antibacterial surfaces* are ways to reduce biofilms by the use of TiO_2_ and its photocatalytic activity, ensuing ROS generation. Immobilization of surfaces by AgNPs is applied on anti-adhesion surfaces and structured arrays [92, 93]. Collusive with biofilm is quorum sensing, which is used by bacteria to maintain proximity and communicate in order to synchronize virulence causing gene expression [94].

## Antimicrobial mechanism of action of NPs

Nanomaterials benefit from their controllable and nanosize structures in comparsion to that of bacterial components [95]. High surface area-to-volume ratio ensures a strong surface chemistry in terms of multivalent interactions with bacterial cells or functionalization for a specific charge or targeted delivery. The forces that majorly dominate the nano-bio interface are van der Walls forces, electrostatic forces, hydrophobic interactions and receptor-ligand interactions. Nanomaterials act along multiple simultaneous or correlated bactericidal mechanisms as described below.

### Disruption of bacterial cell wall

Molecular architecture of bacterial cell envelop is the major physical barrier for any antimicrobial. Lipoteichoic/teichoic acid makes diffusion of highly hydrophobic antibiotic moieties across this envelop unfathomable. Materials with positive potential can bind selectively to bacterial surfaces with higher negative potential than mammalian cells. Physical contact of nanomaterials having a proper equilibrium between cationic charge and hydrophobicity (amphiphilicity) accomplish stupendous antimicrobial property with low levels of cytotoxicity and haemolysis [96].

Nanomaterials get anchored electrostatically onto the bacterial envelops and change their membrane potential leading to the depolarization and loss of membrane integrity. As the physical barricade is disrupted, transport discrepancy, respiration impairment and intrusion in metabolic pathways are followed resulting cell death. Typically, graphene-based NPs, regarded as “nano-knives” or MoS_2_, MnO_2_ with sharp edges are known to physically disintegrate bacteria cell wall [97]. AgNPs cause jamming of oxidative phosphorylation due to dissipation of proton motive force as a result of anchoring into the bacterial membrane [70, 98]. Fullerenes kill bacteria by physically rupturing their cell wall integrity [99]. Fortification of bacterial resistance against therapeutics that cause damage to the cell wall is limited thus, making these approaches an attractive strategy for long-term usage.

### Generation of ROS

ROS are intracellularly originated secondary metabolites of oxidative metabolic activity of a bacterial cell. The cellular ROS levels are maintained by endogenous antioxidant defence system. An excessive production of ROS or redox disequilibrium causes oxidative stress causes the destruction of membrane fatty acid, and macromolecules resulting in peroxidation of lipid molecules, oxidation of proteins which cause inhibition of enzymes and DNA/RNA damage leading to mutation/killing in bacteria.

Nanomaterials can produce four different types of ROS with diverse levels of dynamics and activity—hydrogen peroxide (H_2_O_2_), singlet oxygen (^1^O_2_), superoxide radical (O_2_^−^), and hydroxyl radical (·OH). Nanomaterials, such as carbon nanotubes (CNT), fullerenes, TiO_2_, ZnO, CeO_2_ and AgNPs, induce oxidative stress as a major mechanism of their bactericidal property. Major causes for ROS development are (i) pro-oxidant functional groups on the ultra-reactive surface, (ii) multivalent surface of NPs due to involvement of transition metal ions in active redox cycling involving Fenton-type or Haber–Weiss reactions, and (iii) cellular internalization of NPs leading to activation of NADPH oxidase or mitochondrial respiration. Light-induced ROS production is witnessed in AuNPs and TiO_2._ These are photocatalytic when irradiated with visible light, near UV or UV, proving to exert effective antibacterial potency against spores of *Bacillus* [100, 101]*.* AuNPs have been found to impose antibacterial effect against *Corynebacterium pseudotuberculosis* via ROS generation leading to vacuole formation [102].

Fe_3_O_4_@TiO_2_ core shell magnetic NPs are effective against MDR *S. pyogenes* and MRSA via cascade of bactericidal effects through the generation of ROS that increases the cell porosity and expulsion of intracellular components [103]. Scanning electron microscopy revealed that nano-ZnO changed the spiral shape of *Campylobacter jejuni* into spherical shape leading to loss of membrane integrity [104].

### ROS-independent oxidative stress

ROS-independent oxidative stress is also a major pathway for bacterial killing. In the absence of light and oxygen, nC_60_ exerts its antimicrobial actions without ROS production [105]. For example, nC_60_ directly interacts with cell membrane and disrupts the electron transport system [99, 106]. ROS-independent cytotoxicity exhibited by single walled carbon nanotubes include glutathione disulfide oxidation [107]. Pt-Au bimetallic NPs exert ROS-independent membrane depolarization [108].

### Photo-induced antibacterial action

Photo-induced methodologies using nano substrates are being exploited due to minimal invasiveness, localized treatment modality with minimal toxicity. Upon photoexcitation, photothermal agents (PTAs) can transform optical energy into thermal energy upon non-radiative relaxation to the ground state, leading to irreversible damage to bacteria via membrane rupture, protein denaturation and overall collapse of intracellular components [109].

The local hyperthermia is predominantly caused by thermal conduction or generation of vapour bubbles as a consequence of strong heating. The biologically transparent window of near-infrared (NIR) light from 700 to 950 nm is used for photothermal therapy (PTT) due to deep tissue penetration and low absorption by water and haemoglobin. CNTs were the first carbon-based nanomaterial to be exploited for photothermal bacterial killing owing to their high photothermal conversion efficiency and low fluorescence quantum yield [110]. PEG-modified core–shell gold nanorods layered double hydroxide nanomaterials (GNR@LDH-PEG) showed about 99.25% and 88.44% of bacterial killing rates for *E. coli* and *S. aureus*, respectively, with 5 min of NIR irradiation attributed to electron deficiency on the gold surface [111]. Wang et al. constructed antibacterial coating on Ti plates by hybridization of MnO_2_ with CS modified AgNPs [112]. Upon photoexcitation over NIR irradiation, MnO_2_ NPs were able to produce sufficiently high local heating with photothermal conversion efficiency of 30.79% and exceptional photostability. Combined effects of MnO_2_ with AgNPs were also observed, aiding to the antibacterial property of the nano-construct [112]. Metal sulphide nanomaterials are also explored for antibacterial PTT. For example, magnetic MoS_2_, surface functionalized with chitosan, cause bacterial crosslinking along with their photothermic sterilization [113].

Photo-induced generation of ROS causes oxidative stress, which eventually lyse bacterial cells by disrupting the protein function and inhibiting the activity of certain periplasmic enzymes [114]. Under light activation, at energies greater than or equal to the band gap, stimulated electron transduction from valence band to conduction band results in the formation of electron (e^−^)-hole (H^+^) pair on the surface and intrinsic region of the semi-conducting materials. H^+^ adheres to the surface of nanomaterial and on interaction with H_2_O or OH^−^ gets oxidized to ·OH free radical. ·OH adheres on the surface and electronically interacts with molecular O_2_ that is subsequently reduced to superoxide free radical O_2_^−^ [115, 116]. These generated ROS are highly oxidative and disturb the normal physiology and morphology of bacteria under UV or visible light illumination. TiO_2_ NPs are a classic example of photocatalyst antimicrobial agent when irradiated with near-UV or UVA [117]. The efficiency of TiO_2_ NPs depends on band gap engineering and thickness of microbial surfaces. Magnetic nanoprobe comprising of Fe_3_O_4_@TiO_2_ core–shell nanostructures are used for photo killing of MDR *S. pyogenes* and MRSA under UV irradiation [103].

Photo-induced ROS employs appropriate excitation wavelength and O_2_ as external stimuli factors that selectively activate photosensitizer (PS) at the site of infection producing cytotoxic ROS such as ^1^O_2_ and ·OH [118]. Photodynamic therapy (PDT) essentially operates via two pathways—type I and type II. Upon light sensitization, PS in the ground electronic state transits to the short-lived but higher energy singlet excited state (S_1_). Subsequently, S_1_ undergoes inter-system crossing to a longer-lived excited triplet state (T_1_) [119, 120]. In type I PDT, the triplet excited state of PS interacts with biological macromolecules to generate ^1^O_2_ and ·O_2_^−^ mediated through electron transduction, breaking the structural integrity, and altering ionic permeability of the bacterial membrane [121]. In type II PDT, the triplet excited state transfers energy to ^3^O_2_ to produce lethal ^1^O_2_ species, which in turn interact with immediate biological components causing irreversible oxidative damage to DNA, enzymes, and other cellular components of bacteria [122]. Singlet oxygen has a short lifetime in cellular surroundings and a very small radius of action of ~ 0.2 μm, thus creating a contained response without affecting distant cells. Chlorin e6 (Ce6) loaded into silica NPs showed high antibacterial efficiency against MRSA [123]. Photostability of Ce6 was enhanced with high ^1^O_2_ retention capacity upon light irradiation [123]. A lipase sensitive transfersomal nanocarrier with high skin penetration efficiency was tested against *Propionibacaterium acnes*. Pheophorbide A (PheoA), in absence of lipase gets self-quenched due to intramolecular interactions but PheoA loaded liposomes caused 99% *P. acnes* elimination due to lipase mediated disruption of transfersomes, the quenching of PheoA was reduced considerably [124].

### Controlled release of NP metal ions

Under the influence of external stimuli, the controlled release of metal ions is attributed to the large surface area of NPs. These metal ions get absorbed into the cell membrane leading to direct interface with functional groups of essential proteins and nucleic acids. They infiltrate bacterial cell facilitated by ion channels and biological pump, accumulate above tolerable range causing bacterial cell death [125]. Zn^2+^ ions are known to bind strongly with –SH functionality of cell membrane, leading to loss in membrane integrity [126]. Silver ions leads to protein coagulation aided by Coulomb gravity via a process called biosorption [127]. The presence of Zn^2+^ in addition to Ag^+^ reinforces the bactericidal propensity, providing more active sites resulting in faster adsorption speed and capacity [128]. The solubilization is essential for nanomaterials for production of toxic Zn^2+^ or Ag^+^ which increases oxidative stress through Fenton’s reaction. Super-magnetic iron oxide releases Fe^3+^/Fe^2+^ which can easily penetrate the cell membrane and interferes in transduction of transmembrane electron to produce O_2_^−^, which damages the iron clusters [129]. But the uptake of ZnO NPs is better than Zn^2+^, which directly impact the antibacterial potency [130]. This can be explained by the uptake of nanomaterials mediated through endocytosis and assist the entry of toxic ions via “trojan horse effect” circumventing the defence mechanisms of cell.

### Disruption of protein synthesis

Mindfully engineered nanomaterials arrest bacterial growth and eventually kill bacteria via down regulation of genes, disruption in protein synthesis, and oxidative damage to the DNA [131]. Transcriptomic and proteomic studies to understand the mechanism of action of AuNPs identified two main processes. First barred the merging of ribosomal subunit with the t-RNA and the second, reduction in cellular ATP levels due to disintegration of membrane integrity and loss of ATPase activity [132]. Au-superparamagnetic iron oxide NPs have strong affinity for disulphide bond of bacterial proteins, which affects metabolism and redox homeostasis of the cell [133]. Spores of *B. cereus* are inhibited by *S*-nitrosothiol, which is produced by nitric oxide (NO) releasing NPs; where NO reacts with thiols to produce *S*-nitrosothiol, which nitrosylate thiol residues on bacterial proteins [134]. NO releasing silica NPs are non-toxic to mammary fibroblasts and are also known to stimulate the host immune responses that shrug off the microbial load [135].

### DNA damage

Ag^+^ ions intercalate DNA strands due to complexation with nucleic acid aided by Coulomb interactions and breaking the H-bonds that hold purine and pyrimidines base-pairs together [136]. AgNPs exhibit genotoxic potential by obstructing bacterial DNA unwinding and transcription, infringement of DNA chains and causing chromosomal irregularity [137]. Deamination of cytosine, guanine and adenine is mediated by N-nitrosating agents formed by auto-oxidation of NO [138]. DNA strand breaks are formed by peroxynitrite and NO_2_ and therefore, NO releasing therapies have been proposed as promising antimicrobial agents [134].

### Gene regulation

AgNPs cause upregulation of antioxidant genes and genes encoding metal transport, metal reduction and ATPase pumps [139]. In *E. coli,* polymer-coated AgNPs were shown to downregulate the *aceF* and frdB genes that are responsible for tricarboxylic acid cycle and *gadB*, *metL*, and *argC* genes for amino acid metabolism [140]. MgONPs upregulated the expression of weak-thiamine ester-binding and riboflavin metabolic genes and downregulated the expression of metabolite coding genes, disrupting physiological functioning of bacteria [141].

### Increased retention of aggregated NPs in biofilms

The large aggregated AgNPs have been shown to exhibit better penetration ability in infected areas and longer retention in the bacterial biofilms, disrupting biofilm formation and effectively eliminating bacterial populations [70]. Aggregated AgNPs would also exhibit longer retention periods in tissues and the exocytosis from the cells would be relatively slower than small non-aggregated particles, adding to their therapeutic implications [70].

**Fig. 4:**
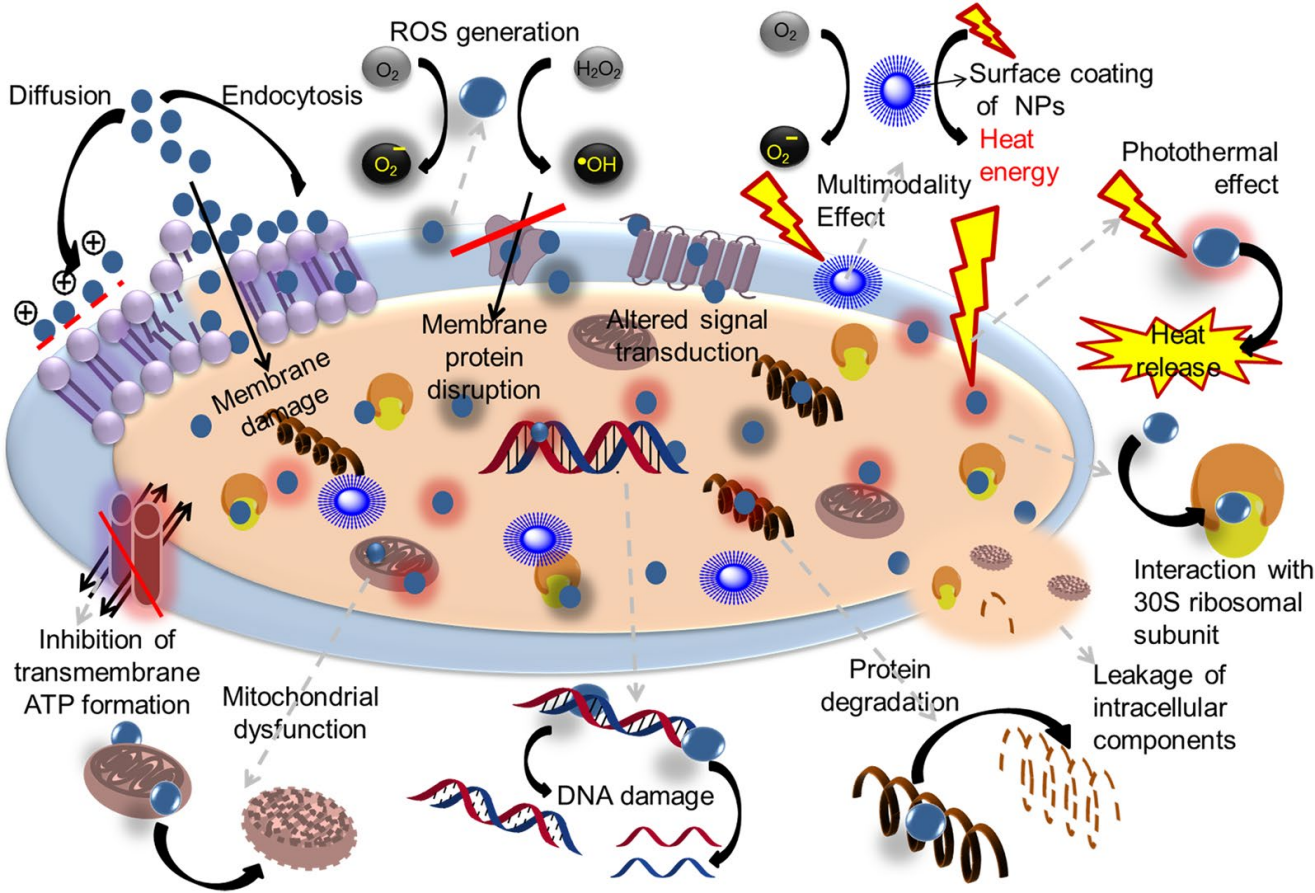
The nano armamentarium to combat AMR. NPs can attach the microbial cell wall and penetrate it, thus causing structural changes in the cell membrane permeability leading to cell death. NPs target at the cell membrane, leading to dissipation of proton motive force thus blocking the oxidative phosphorylation. NPs have ability to produce ROS that can cause DNA damage leading to cell death. They can impel the shape and function of cell membrane, interact with DNA, ribosomes, lysosomes, and enzymes, promoting fluctuations in cell membrane permeability, oxidative stress, heterogeneous alterations, enzyme inhibition, electrolyte balance disorders, protein deactivation, and changes in gene expression leading to bacterial cell death

Figure 4 summarises possible mechanisms of action of NPs against bacteria.

### Antibiotic nanocarriers

In addition to directly targeting bacteria, certain nanomaterials are being exploited as “antibiotic nanocarriers” to encapsulate traditional antibiotics in order to improve pharmacokinetics, stability and bioavailability, and enhance bacteria eradication [142, 143]. This approach improves the efficacy of antibiotics while reducing potential toxicity. Furthermore, nanocarriers decrease the apparent volume distribution, in turn allowing maximum tolerated dose and causing bacterial cell death at much lower antibiotic concentrations and thus expanding the spectrum of action against MDR bacteria. Various biopolymers including proteins, carbohydrates and lipids have been used to synthesize nanocarriers with promising antibacterial activities [144]. For example, linalool-functionalized hollow mesoporous silica spheres efficiently improved the bactericidal activities of the organic component against both Gram-negative (*E. coli*) and Gram-positive (*S. aureus*) bacteria by breaking the cell membrane structure [145]. A nano-delivery system constructed by self-assembly of antibacterial berberine and rhein phytochemicals showed good biocompatibility and safety profiles and strong inhibitory effects on *S. aureus* biofilm [146]. Strategies to encapsulate multiple antibiotics in a single nanocarrier for obtaining a synergistic effect are also being investigated. The delivery of antibiotics using nanocarriers results in highly targeted delivery of huge concentrations of antibiotics to the bacterial cells, increasing the overall efficacy and therapeutic potential. For example, the nanocarrier system, developed using carboxyl-modified mesoporous silica nanoparticles carrying vancomycin and polymyxin B, showed improved biocompatibility and synergistically high antibacterial efficacy [147].

## Potential acquisition of bacterial resistance to NPs

NPs attack pathogens with multifunctional mechanisms that are diverse from the contemporary antibiotics. Thus, numerous mutations are required in bacteria to develop resistance toward NPs. However, resistance of microbes to NPs is still a concern [148, 149]. The common resistance patterns encompass ion efflux pumps, electrostatic repulsion, adaptation of biofilms, expression of extracellular matrices, enzyme detoxification, followed by volatilization and mutations [150, 151]. Nano-resistance changes the shape of bacteria and modulates the expression of membrane proteins, which reverses on removal of NPs [152]. The formation of biomolecules corona reduces the binding of metal-based NPs to bacteria, thus imparting resistance [153]. Although rare, but resistance has also been reported against Cu, Au and Ag NPs which can be attributed to high expression of efflux pumps, and alteration in membrane permeability [148, 149, 154].

### Defense mechanism against various NP sizes

Increased clinical use of AgNPs is raising concern as resistance has been detected in *K. pneumoniae* and *E. cloacae* [154]*. E. coli* was found to be resistant to AgNPs due to NP aggregation by the altered production of flagellin, diminishing the toxicity of AgNPs [149]. Agglomeration and deactivation of NPs are the mechanisms used by bacteria to prevent the effect of NPs. *E. coli* acclimatize to AgNPs by releasing ECS. ECS amends the size and zeta potential of NPs through their agglomeration [155]. Bacteria exposed to sub-lethal concentrations of AgNPs showed enhanced resistance against these NPs due to mutations that caused the downregulation of porins, inhibiting the entry of NPs in bacteria [156]. *P. putida* changes the conformation of unsaturated fatty acids of its membrane, thus modifying the fluidity of the membrane making it less permeable, hence, blocking the cellular entry of NPs and metal ions [157].

### Defence mechanisms against surface charge

Charge on the surface of various metal/metal oxide NPs is important for antimicrobial activity [158, 159]. Bactericidal action of MgONPs against endospores, Gram-positive and Gram-negative bacteria is due to electrostatic attraction of positively charged NPs with negatively charged bacteria [160]. Microbes can modulate electrical charge on their surfaces, which can ward off the NPs. Antibacterial potency of positively charged AgNPs is better against Gram-positive (*S. pyogenes*, *S. mutants*, *S. aureus*) and Gram-negative (*P. vulgaris* and *E. coli*), in comparison to neutral or negatively charged NPs. However, some bacteria can develop resistance to cationic antimicrobial peptides (CAMP) by changing the surface charge through modification of phospholipids [161].

Envelope stress response (ESR), in both Gram-positive and Gram-negative microbes, safeguards the integrity of microbial cell envelope [162]. It incorporates D-alanine in Gram-positive microbe, decreasing the overall negative net charge and thus protecting against positively charged NPs; whereas in Gram negative bacteria, it adds phospho-ethanolamine (PEA) in lipid-A component of the LPS, which increases positive charge on bacteria [163].

### Defence mechanisms against metal ions release

Industrial production of NPs for electronics, computers, food, domestic and automotive products, leads to their accumulation in the environment. These transform the NPs due to change of physicochemical and antimicrobial properties [164]. Transformation can lead to altered dissolution and ion release rate due to ligation, aggregation, redox reaction (oxidation/ interaction with natural organic matter of zero-valent Fe NPs at aerobic condition inhibits antimicrobial properties), adsorption of biomolecules and biotransformation [165, 166]. This allows bacteria to adapt to metal ions through the efflux systems (expressed from metal resistance genes). For example, interaction of *P. aeruginosa* with CuONPs showed upregulation of cation diffusion facilitator (CDF), RND membrane fusion protein family, and OM factors. These make the bacterial CzcCBA efflux system, transporters, and P-type ATPase efflux complexes [167] that facilitate the efflux of metal ions such as Co^2+^, Zn^2+^, Cd^2+^, Ni^2+^, Cu^+^ and Ag^+^. Cation diffusion facilitators (CDF family) form the second line of defence that mediates the efflux of Zn^2+^, Co^2+^, Ni^2+^, Cd^2+^, and Fe^2+^ [168]. P-type ATPases forms the third line of defence and efflux is carried out by ATP hydrolysis. It arbitrates the efflux of H^+^, Na^+^, K^+^, Mg^2+^, Ca^2+^, Cu^+^, Ag^+^, Zn^2+^ and Cd^2+^ [169].

Other mechanisms include pigment production, intra/extra cellular sequestration, morphology alteration bioprecipitation, and biotransformation or enzymatic detoxification [170]. Bacteria also have metal-resistant operons; ion-sequestering proteins like SilG gene in sil operon [171]. Release of pigments by bacteria reduces their exposure to the ion of NPs thus reducing antimicrobial activity, like *P. aeruginosa* produces pyocyanin which protects bacteria by reducing Ag^+^ to Ag^0^ [172]. Through biotransformation, bacterial enzymes transform toxic ions to non-toxic forms [173]. For example, *Lactobacillus bulgaricus* enzymatically transforms the chitosan-modified selenium NPs into organic seleno-compounds like SeCys2 and SeMet [174]. Bacteria can change their morphology resulting in minor bactericidal effect of NPs. *E. coli* becomes oval and small to resist ZnONPs. Aforesaid mechanisms are regulated by metallo-regulatory and metal homeostasis systems in microbes [175].

The extensive use of metal/metal oxide NPs can stimulate the co-selection and co-expression of antibiotic resistance genes. Exposure of *E. coli* at sublethal concentrations of ZnONPs and TiO_2_NPs facilitated the conjugative transfer of antibiotic resistance plasmids as NPs increases the permeability of cell membranes [176, 177]. ESR also regulates the defence mechanism against ion release, electrical charge, and size of various metal/metal oxide NPs. In Gram-negative bacteria, ESR has an alternative sigma factor (RpoE or σE), three different 2-component regulatory systems [pilus expression (Cpx) response, regulation of capsular synthesis (Rcs) phosphorelay and bacterial adaptive response (Bae)] and phage shock protein response (PSP). RpoE regulates the cell envelope biogenesis, protein folding and degradation, and cell envelope modification. It controls alginate production in *P. aeruginosa*. RpoE modifies LPS through PhoPQ regulon and MicLs RNAs, which increases the resistance to antibacterial molecules like CAMP and NPs. Cpx regulates the protein export systems associated with virulence. Rcs reaction (second of the three different 2-component regulatory systems) can disrupt cell envelope and control the production of macromolecular envelope structures and its homeostasis. Bae regulates the expression of efflux pumps while PSP sustains proton motive force and determines localized secretin toxicity. ESR in Gram-positive bacteria has 3 systems which regulate the cell wall metabolism, cell envelope charge, and expression of efflux pumps. The first regulates retort to explicit toxic stimuli. Second is activated by dangerous agents existing in the cell wall. Third controls the cell envelope integrity [178].

### Defence mechanisms against ROS and oxidative stress

Metal ions affects respiration and scavenging mechanisms resulting in accumulation of singlet oxygen, OH radical, H_2_O_2_, superoxide, and other ROS. ROS can cause damage to the internal components of the bacteria such as structural proteins, organelles, enzymes, DNA, respiratory chain and scavenging mechanisms [179]. ROS at sublethal concentrations fuels defence mechanisms in bacteria, a process called as hormesis [180]. First level of adaption through hormesis is enzymatic or short-term response through expression of ROS scavenger enzymes creating balance in bacteria for few seconds or minutes. Second level is long-term adaptation which consists of two sub-levels: Transcriptional and genomic. At transcriptional level, upregulation of antioxidant mechanisms takes place within hours to days [181]. Activation of DNA damage repair mechanisms (excision repair, homologous recombination, and translation DNA synthesis) takes place at genomic level. This creates genome plasticity by repairing the incorrect bases in DNA resulting in resistance to metal/metal oxide NPs [182]. Two regulons: SoxR and OxyR activate the genes responsible for resistance against oxidative stress. OxyR responds to stress induced by H_2_O_2_, and SoxR responds to superoxide anion [183].

### Defence mechanisms of biofilms

Biofilm is a union of varied microbes within a matrix of extracellular polymers (lipids, DNA, polysaccharides, and proteins), which acts a barricade for NPs to contact bacteria and set of resistance mechanisms are introduced due to various microbial species [184, 185]. ECP modulates size, surface charge, shape, and concentration of NPs. Biofilms need higher concentrations of NPs than planktonic cells because of grouping of microbes. Biofilms of pore size 10–50 nm retain NPs of > 10 nm size, this trapping do not let NPs interact with microbes present deep inside the biofilm, thus rendering NPs ineffective [186]. Whereas in mature biofilm, the pore size is smaller this does not allow the penetration and diffusion of NPs [187]. Sublethal concentrations of NPs to biofilms can generate hormesis.

## Clearance and elimination of nanomaterials

The biodegradation and removal of NPs through urinary and biliary pathways is largely low, leading to persistent tissue retention, amassing in liver and spleen and hence augmented toxicity [188, 189]. Charge and size of NPs determine their opsonisation by serum proteins. In this process, the effective size or hydrodynamic diameter (HD) of the NPs can be modulated. Pore size of endothelium is 5 nm, and NPs with HD < 5 nm can get balanced with the extravascular extracellular space (EES), as vascular endothelial layer acts as dynamic and semi-selective barrier. Thus, large sized NPs cannot freely move in the endothelium hence continues prolonged circulation and toxicities [189]. HD has a crucial part in renal clearance as there is a converse relation between HD and rate of glomerular filtration. NPs of HD < 6 nm undergo glomerular filtration and get proficiently filtered but that of > 8 nm do not. Renal filtration rate is determined by charge as well because interactions of charged NPs and serum proteins (improved protein adsorption), leads to increased HD. Cationic, neutral, and anionic is the order in which these NPs get filtered through glomerulus [190]. Prospective communications between charged capillary walls of glomerulus and NPs also monitor the renal filtration rate [191, 192]. NPs (10–20 nm) cannot use renal filtration, but hepatobiliary system. Liver clears NPs through phagocytosis by Kupffer cells followed by intracellular degradation and eradication. NPs which escape these removal processes retains in the body.

## Dose optimization and toxicity of antimicrobial nanomaterials

NPs and their toxic by-products can cause lysis of RBCs and impede blood coagulation pathways [193]. In vitro studies depict high toxicity of AgNPs along with dysfunction of vital organs like; lungs, spleen, liver, colon, bone marrow, and lymphatic system in vivo (and via intravenous and inhalation use of NPs in patients) due to their accumulation [194]. Ag can leach out from wound dressings and have been tested in urine and blood. Al_2_O_3_NPs interact with various inter/intra cellular biomolecules and cause neurotoxicity [195]. NPs of CuO cause oxidative distress (prompt nephro- and hepatotoxicity) and ZnO/TiO_2_ roots DNA damage [196].

The judgement for optimum dose is fundamental for therapeutic targets and curtailing toxicity for clinical translation. Realistic and suitable doses should be researched as the statistics from in vitro and in vivo studies may not be directly translated to patients. This brings concern to the table, the interaction of NPs with human tissues/organs and its clinical translation and administration as therapeutic.

## Nanomaterial-based vaccines

Immunization by vaccine remains the most effective method to provide protection against infectious diseases [197]. However, clinical applicability of many new potential vaccine candidates is limited due to low immunogenicity and inability to stimulate an effective long-lasting immunity [198, 199]. In particular, subunit vaccines are less immunogenic and often fail to evoke desirable immune reactions. These vaccines require appropriate adjuvants (immune stimulating agents) and innovative delivery systems to increase immunogenicity, intensify innate and adaptive immunity, and provide a long-term memory response; but concerns remain about their safety and tolerability [200]. Several NPs have shown to stimulate immune responses by multiple mechanisms, offering alternatives to currently used adjuvants and vaccine delivery systems [201]. For example, NPs allow the expression of proteins of interest in the correct conformation on their surfaces thus promote a precise immune reaction. NPs are used as delivery vehicles that guard antigens from degradation. In this context, nanomaterials with defined composition, tailorable structures, tunable immunostimulatory properties, and progressive engineering design are excellent candidates for vaccine development to effectively prevent and manage bacterial infections.

Formulation, “cold-chain” storage, stability, route of administration, and long-distance transportation of vaccines are other technical challenges for vaccine development and effective vaccination programs. In this regard, engineered nanomaterials with varying origins, sizes, shapes, surface properties, and biological functionalities, serve as desirable delivery systems, protect vaccine/antigens from degradation, enhance antigen processing and presentation, facilitate vaccine/antigen uptake by professional antigen-presenting cells (APCs), and improve the stability of vaccines [202]. For example, macrophages preferentially ingest anionic particles [203]. Cationic NPs, such as chitosan, are mucoadhesive that allow them to remain in the mucus for longer duration and interact with mucosal immune cells [204, 205]. In addition, nanomaterials offer routes to improve adjuvants’ function, dose reduction by controlled release of antigen/adjuvant near or within APCs, and minimize undesirable systemic or local effects, such as excessive inflammation or toxicity [206–208].

Nanotechnology offers great platform to design novel modern vaccines as well as facilitate their global implementation. Several nanomaterial-based vaccines to control bacterial infections have been approved for human use and there are many being tested in pre-clinical or clinical trials (Table 2). These vaccines use diverse range of nanomaterials, including polymeric materials (e.g., chitosan), polyesters (e.g., PLGA), polyamides (e.g., gamma polyglutamic acid), polyelectrolyte multilayers, and dendrimers. Their main applications in vaccine include transport and deliver varied peptide, nucleic acid, and protein antigens and adjuvants [206]. Antigen can be delivered to the target cells by either encapsulation (this inhibits premature antigen deprivation and attains persistent release) within NPs or by adsorption on the particle surface (this stabilizes the antigen and simplifies receptor-mediated uptake by APCs) [201, 202]. The particulate nature of NPs bestows them with the capacity to manipulate, enhance and optimize antigen density, and stimulate innate immune response while APCs internalize and process the antigen [209]. Nanomaterials can be engineered to simultaneously co-deliver multiple antigens and adjuvants, which is particularly important for controlling the quality and strength of immune responses. They can be tuned to skew the immune polarization towards important subtypes, such as elicitation of Th1 responses. All these fitting properties of NPs can improve vaccine delivery and efficiency when equated to other conventional delivery and adjuvant systems. Thus, NP-based vaccine formulations are an advantageous, favorable and safe strategy to vaccine development to tackle AMR.

**Table 2:** Selected nanomaterial-based vaccines against bacterial infections

Antigen	Nanocarrier used	Disease	References (year)
Antigenic protein	PLGA nanospheres	Anthrax	[210] (2018)
DNA encoding T cell epitopes of Esat-6 and FL	Chitosan nanoparticle	Tuberculosis	[210] (2018)
Mycobacterium lipids	Chitosan nanoparticle	Tuberculosis	[210] (2018)
Polysaccharides	Liposomes	Pneumonia	[210] (2018)
Bacterial toxic and parasitic protein	Liposomes	Cholera and malaria	[210] (2018)
Fusion protein	Liposomes	*H. pylori*	[210] (2018)
Antigenic protein	Nano-emulsion	Cystic fibrosis, Anthrax	[210] (2018)
Mycobacterium fusion protein	Liposome	Tuberculosis	[210] (2018)
Flagellin protein	AuNPs	*Yersinia pestis*, *S. pneumoniae*	[211] (2012)
Antigenic protein	Cationic liposome-based, stabilized with synthetic glycolipid (CAF01)	Tuberculosis	[212] (2014)
Plasmid DNA encoding BoNT heavy chain (Hc)	PLGA	*Clostridium botulinum*	[213] (2016)
Capsular polysaccharide serotype 14 and T-helper peptide, ovalbumin 323–339 peptide	AuNPs with branched tetra-saccharide unit b-d-Galp-(1–4)-b-d-Glcp-(1–6)-[b-d-Galp-(1–4)-]b-d-GlcpNAc-(1–)	*S. pneumonia*	[214] (2012)
LomW and EscC	AuNPs	*E. coli (EHEC)*	[210] (2018)
Listeriolysin O (91–99) and glyceraldehyde-3-phosphate-dehydrogenase (1–22) peptide	AuNPs	*Listeria monocytogenes*	[210] (2018)
Hemagglutinin and FIgL	AuNP coated with antigenic capsular LPS	*Burkholderia pseudomallei*	[215] (2017)
N-terminal domains flagellin (1–161)	AuNPs	*P. aeruginosa*	[216] (2016)
Monosialotetrahexosylganglioside (GM1), host receptor for cholera toxin	PLGA	*Vibrio cholerae*	[217] (2018)
Serogroup B	OMV-based vaccine	*N. meningitidis*	[218] (2004)
Membrane proteins	Double-layered membrane vesicles	*P. aeruginosa*	[218] (2004)
Immunodominant antigens (Ag85A & ESAT-6) and IL-21	Fe_2_O_3_ coated plasmid DNA TB vaccine	*M. tuberculosis*	[219] (2012)
Recombinant fusion protein (M72)	Liposomes	*M. tuberculosis*	[220] (2018)
Heat-induced OMV from enterotoxigenic *E. coli*	Poly(anhydride) NPs	*E. coli*	[221] (2022)

### Types of nanomaterials used for vaccine production

Various types of NPs of carbon, gold, polymers, dendrimers, liposomes, and immune-stimulating complexes are used to stabilize and deliver vaccines (antigens and adjuvant) that can effectively stimulate immune responses, such as the production of cytokines and antibody responses [222, 223]. However, emerging AMR bacteria create a challenge for new live-attenuated (first generation), synthetic (second generation), DNA/RNA (third generation), and subunit vaccines [224–226]. In this context, NPs provide a viable approach to vaccine delivery, improving vaccine efficacy with slow release, easy antigen uptake, and induction of humoral and cellular responses.

### Mechanism of improved immune response by nanomaterial-based vaccines

A major facet in the engineering of nano-vaccines against bacterial diseases implicates their delivery and interaction to key immune cells, like macrophages, dendritic cells, neutrophils, B and T cells. It also ensures that post vaccine-associated antigens have been processed. Multiple factors like, size and shape, in vivo durability, number of antigen copies on/within nanomaterial, precise co-delivery of adjuvants, conformation/orientation of antigens, or complement activation, impact the vaccine delivery to various tissues, with sizeable impression on the quality and strength of upraised immune responses [227].

Encapsulation of antigens within nanomaterials can upsurge the persistence of antigens at injection site in lymphoid cells or in APCs, which in turn can enhance its immunogenicity. This procedure of producing high affinity antibodies to target is dire for causing immunity against infections. For durable cellular immune response, antigens need to be internalized, processed, and presented efficiently by APCs. For efficacious cross-presentation, antigens must escape to the cytosol, for which nanomaterials sensitive to endolysosomal pathway milieu have been travelled [228]. The precise delivery of nano-vaccines to B, T, follicular dendritic cells, and subcapsular sinus macrophages is highly sought for vaccine design. Physical properties, such as charge, shape, and flexibility of NPs influence lymphatic drainage [229]. Hydrophilic polymers have been found to assist transfer of NPs across the mucus layer [230]. Also, cationic NPs are mucoadhesive, permitting them to be reserved in mucus and to interact with mucosal immune cells. Cationic polysaccharide, chitosan has been employed in mucosally delivered vaccine NPs, including TB [231].

Nanomaterial vaccines also mediate improvement of adjuvant functions and minimize negative systemic/local effects, like inflammation or toxicity. Moreover, some NPs have intrinsic adjuvant properties, even without augmentation by TLR ligands or other adjuvants, which might occur by complement activation, inflammasome signalling or B cell activation [223]. These properties of nanovaccines are beneficial because they can limit inflammation and toxicity arising from other adjuvants and simplify the formulation and dosing of a vaccine.

## NP-investing companies and clinical trials

Many companies are considering vaccines as favorable preventive approach to handle AMR because they can be used directly as an inhibitory tool against deadly pathogens. Vaccines can reduce the use of antibiotics by decreasing the infection symptoms, which trigger the antibiotic consumption [232]. Most importantly, vaccines can stop the increment of bacterial numbers, and thus reduce the chances of AMR mutations [233]. The advent of recombinant DNA and glycoconjugation techniques drove the possibility of development of better vaccines against resistant pathogens such as *H. influenzae* type B, *Pneumococcus*, *Meningococcus*, group B *Streptococcus*, *E. coli*, and *Shigella*) [234, 235].

The successful cases of nanobiotics in clinical applications are witnessed in recent years. Liposomal amikacin for inhalation (ARIKAYCE®, developed by Insmed Incorporated) is a unique formulation that encapsulates aqueous Amikacin in charge-neutral liposomes composed of dipalmitoyl phosphatidyl choline and cholesterol [236]. This formulation has been approved by USFDA for treating patients with *Mycobacterium avium* complex lung disease refractory to conventional therapy and with limited or no alternative treatment options [237]. Liposomal encapsulated ciprofloxacin (rapid-release formulations (Lipoquin™ or ARD-3100) and slow-release formulations (Pulmaquin™ or ARD-3150), developed by Aradigm Corporation for non-cystic fibrosis bronchiectasis colonized with *P. aeruginosa* are commercially available [237]. Moreover, many nano-antibiotics are in clinical use, such as, mupirocin, polymyxin B, fluconazole, gentamicin, and so on. Prevnar, a pneumococcal vaccine developed by Wyeth, consists saccharides of capsular antigens of seven serotypes of *S. pneumoniae* conjugated to mutant diphtheria toxoid CRM197 [238]. With the unremitting efforts of researchers, the prospect of clinical nano-antibiotics/nanomaterials would be more hopeful.

## Challenges of the NPs for clinical applications

The foremost contests associated with clinical translation of nano-vaccine/nanomedicine are safety, biological concerns, biocompatibility, intellectual property, laws/regulations, time and cost-effectiveness, which discourage the usage of nanomaterials in the present markets [239, 240]. Therefore, one should consider nano-pharmaceutical designs for their physical/chemical stability, biocompatibility/biodegradability, and administration route. Determinations for resolving hindrances of large-scale production, batch to batch reproducibility, high cost, quality control assays (polydispersity, scalability complexities, purification from contaminants, consistency and storage stability, morphology, and charge) should be made [241, 242]. Another challenge is preclinical assessment, for example, toxicity detection (administration and interaction of nanomaterials with biological tissues), in vivo estimation, and understanding pharmacokinetics/and pharmacodynamics of the nanomaterial-based therapeutics and vaccines. It is also important to explore clinical examination for commercialization as invention to market is a difficult path to follow for antibiotics.

As nanomaterials embody countless types of nanostructures, challenges in evolving regulatory protocols, approaches for certifying consistent good manufacturing practice (GMP) production, characterization, safety, and economic design of clinical trial are frequently faced. Since nanomaterial-based vaccines patents and intellectual properties are intricate and universalization of them is desirable, it is important to simplify the path from invention to commercialization to reduce the time and expense required for negotiating collaboration and licensing agreements [243]. Thus, there is a long stretch that needs to be followed before plunging nanomaterials into clinical trials and medicine to control AMR.

## Conclusion and future directions

It is imperative to comprehend the mechanisms by which nanomaterials can influence microbial viability, as many antimicrobial mechanisms of nanomaterials are still ambiguous. For example, majority of research points the antimicrobial potential to ROS/oxidative stress, whereas for MgONPs, the mechanism may not be associated with regulation of metabolism of microbes. Thus, finding the antibacterial mechanisms of various nanomaterials is noticeably applicable in addressing for future research. The absence of consistent criterions is one limitation of current studies on mechanisms of NPs because there is no solitary scheme which accomplishes all the studies for procurement of evidence of antimicrobial mechanisms of various NPs as each type displays discrete antibacterial effects. Thus, a complete comprehensive analysis is recommended to study the probable mechanisms. More in vivo studies should be incorporated as it is inconsistent to evaluate the antimicrobial action of NPs merely through in vitro studies. It is also insisted that toxicity of NPs on human cells especially neurotoxicity should be conducted as the crossing of NPs by blood brain barrier is less explored. Though few studies find that NPs enter the bacterial cells through porins, it would be important to understand detailed mechanism of NP entry into bacterial cells. Since nanobiology is getting increasing attention from scientists, the production of biogenic NPs by use of safe and eco-friendly natural reducing agents should be the basis of research on NPs synthesis. Multiple desirable properties make nanomaterials useful against widespread MDR bacteria. The translation of nanomaterials into clinical applications is a craft that need to be done by researchers in order to come up with the best and active antibacterial therapeutics.

## Abbreviations

AgNPs: Silver nanoparticles
AMEs: Aminoglycoside modifying enzymes
AMR: Antimicrobial resistance
APCs: Antigen presenting cells
AuNPs: Gold nanoparticles
CAMP: Cationic antimicrobial peptides
CDF: Cation diffusion facilitator
EES: Extravascular extracellular space
EPS: Extracellular polymeric substances
GQDs: Graphene quantum dots
HD: Hydrodynamic diameter
HGT: Horizontal gene transfer
LNPs: Liposomes and lipid nanoparticles
LPS: Lipopolysaccharide
MDR: Multi-drug resistant
MRSA: Methicillin-resistant *Staphylococcus aureus*
NO: Nitric oxide
NPs: Nanoparticles
OM: Outer membranes
PBP: Penicillin-binding protein
PDT: Photodynamic therapy
PEA: Phospho-ethanolamine
PLA: Polylactic acid
PS: Photosensitizer
PLGA: Polylactic-co-glycolic acid
PTT: Photothermal therapy
QDs: Quantum dots
ROS: Reactive oxygen species
TA: Toxin-antitoxin
UTIs: Urinary tract infections
